# Numeracy Gender Gap in STEM Higher Education: The Role of Neuroticism and Math Anxiety

**DOI:** 10.3389/fpsyg.2022.856405

**Published:** 2022-05-26

**Authors:** Maristella Lunardon, Tania Cerni, Raffaella I. Rumiati

**Affiliations:** ^1^Neuroscience Area – SISSA, Scuola Internazionale Superiore di Studi Avanzati, Trieste, Italy; ^2^Dipartimento di Psicologia e Scienze Cognitive, Università di Trento, Rovereto, Italy

**Keywords:** mathematics, big five, non-cognitive factors, personality, undergraduate students

## Abstract

The under-representation of women in Science, Technology, Engineering, and Mathematics (STEM) is ubiquitous and understanding the roots of this phenomenon is mandatory to guarantee social equality and economic growth. In the present study, we investigated the contribution of non-cognitive factors that usually show higher levels in females, such as math anxiety (MA) and neuroticism personality trait, to numeracy competence, a core component in STEM studies. A sample of STEM undergraduate students, balanced for gender (*N*_F_ = *N*_M_ = 70) and Intelligent Quotient (IQ), completed online self-report questionnaires and a numeracy cognitive assessment test. Results show that females scored lower in the numeracy test, and higher in the non-cognitive measures. Moreover, compared to males’, females’ numeracy scores were more strongly influenced by MA and neuroticism. We also tested whether MA association to numeracy is mediated by neuroticism, and whether this mediation is characterized by gender differences. While we failed to detect a significant mediation of neuroticism in the association between MA and numeracy overall, when gender was added as a moderator in this association, neuroticism turned out to be significant for females only. Our findings revealed that non-cognitive factors differently supported numeracy in females and males in STEM programs.

## Introduction

The gender gap in Science, Technology, Engineering, and Mathematics (STEM) careers, which almost always penalizes females, is at the core of current educational and economic debates. In Italy, for instance, in 2019 only 37% of the students enrolled in a scientific undergraduate course were females, and the rate of female full professors in STEM was even lower (21%, Morana and Sagramora, 2021). Overcoming the gender gap in STEM is required to achieve gender equality. It is also desirable for economic growth as STEM skills are the most required and the best paid in the labor market (National Science Board, National Science Foundation, 2021). Moreover, reaching gender equality is estimated to increase total European jobs from 850.000 to 1.200.000 units by 2050 (European Institute of Gender Equality, 2017).

In the present study, we focused on the gender gap observed for numeracy competence, as its mastery is predictive of success and retention in STEM careers (Watt et al., 2016; Holmes et al., 2018). Here, we investigated whether such competence is differently supported by two non-cognitive factors—math anxiety and neuroticism—in male and female students at the beginning of a STEM career. Understanding gender differences in numeracy among STEM students may add further details on the reasons why women are penalized in STEM education and career path.

### Gender Gap in Numeracy Competence and Math Anxiety

Numeracy (also known as mathematical literacy) has been defined as a set of knowledge and skills that allow employing mathematics in a variety of private and public contexts, to understand social phenomena, to formulate decisions, and to participate actively in society (Geiger et al., 2015; Organization for Economic Co-operation and Development (OECD); OECD, 2018; Tout et al., 2021). Given that this concept of numeracy refers to abilities that extend beyond basic numerical cognition and formal mathematical contents acquired at school to the broader real-life situations (Gal and Tout, 2014), it is not surprising that better numeracy mastery predicts better life outcomes (Parsons and Bynner, 2005).

This definition of numeracy has been translated into international frameworks, based on which national and international organizations developed assessment programs through specific numeracy achievement tests. The most popular are the OECD’s *Program for International Student Assessment*^1^ (PISA) for high school students, and the *Program for the International Assessment of Adult Compete*ncies^2^ (PIAAC) for adults. High scores on these tests are held to reflect high mathematical competence levels through logical reasoning problems and questions that assess the ability to extract and use information from graphs. Overall, these assessments allow the evaluation of mathematical competencies that are influenced by, but that are not restricted to, basic numerical skills, such as number line estimation, calculation and knowledge about basic arithmetic principles (Morsanyi et al., 2018; Ludewig et al., 2020).

From the first rounds of these numeracy assessment programs, the results clearly showed that females’ achievement in numeracy is lower than males’ (Stoet et al., 2016). This gap shows up earlier in education (Mullis et al., 2020), and grows later on through adolescence (INVALSI, 2019; OECD, 2019) up to adulthood (Borgonovi et al., 2018). However, the gender disparity is not consistent across countries (Else-Quest et al., 2010), with more egalitarian countries, with equal educational opportunities for women and men, displaying a smaller or nonexistent gender gap (Spelke, 2005; Guiso et al., 2008; Cook, 2018). Some recent research failed to find empirical support for gender differences in basic numerical skills and early mathematical cognition (e.g., number and dot comparison, dot estimation and arithmetic, Hutchison et al., 2018; numerosity perception, counting and elementary school-based math concepts, Kersey et al., 2018), suggesting that female and male children and young students enter formal education with the same level of mathematical knowledge. Thus, the numeracy disparity between genders, which is still observed in standardized numeracy tests in various countries, is unlikely to be exclusively associated with pure cognitive factors, such as basic numerical skills. Other non-cognitive factors seem to contribute to its existence. Indeed, performance on cognitive assessment tests, numeracy tests included, is influenced also by personal, social and learning skills (European Commission, 2006, 2018; Borghans et al., 2016).

Among them, math anxiety (MA) is probably one of the most debated factors that underlay the numeracy gender gap. MA is defined as the “feeling of tension and anxiety that interferes with the manipulation of numbers and the solving of mathematical problems in a wide variety of ordinary life and academic situations” (Richardson and Suinn, 1972). Its interference with math performance is well recognized (for reviews, see, e.g., Dowker et al., 2016; Suárez-Pellicioni et al., 2016). Highly math-anxious individuals tend to avoid circumstances involving math whenever possible (Ashcraft, 2002; Beilock and Maloney, 2015), but they also score lower on tests assessing numerical skills. Indeed, recent meta-analyses revealed a significant negative association between MA and performance across different numerical and mathematical tasks (Zhang et al., 2019; Barroso et al., 2021). Notably, previous research consistently reported levels of MA higher in females than males (Betz, 1978; Rubinsten et al., 2012; Ferguson et al., 2015; Hill et al., 2016; Stoet et al., 2016). Thus, women seem to be more likely to suffer the consequences of higher levels of MA, although it is not clear whether MA negatively impacts numeracy performance only in females (Devine et al., 2012; Van Mier et al., 2019) or in both genders (Hembree, 1990; Ma, 1999; Barroso et al., 2021).

Different reasons have been discussed for the higher MA level in females, involving cognitive and non-cognitive factors (for review, see, e.g., Suárez-Pellicioni et al., 2016). As to the non-cognitive factors, several studies showed differences in the way males and females experience mathematics. Attitudes and emotions towards mathematics are more negative in females than males (Muzzatti and Agnoli, 2007; Else-Quest et al., 2010; Pipere and Mieriņa, 2017; Rodriguez et al., 2020; Cohen et al., 2021). For instance, females develop lower levels of academic self-concept (i.e., one’s own evaluation of the one-self; Goetz et al., 2008; Mejía-Rodríguez et al., 2021), self-efficacy (i.e., the belief that personal action and effort can lead to success in mathematics; Louis and Mistele, 2012), and math motivation (i.e., the personal investment and success in solving math problems, Milovanović et al., 2020), and they are more affected by math-related gender stereotypes (Spencer et al., 1999; Appel et al., 2011). All these personal dispositions towards math may contribute to increasing MA and, as a consequence, to impact numeracy performance (see, e.g., Suárez-Pellicioni et al., 2016).

Considering non-cognitive personal factors that are not specific to math domain, gender differences in MA may also be related to a greater personal inclination to feel anxious. Indeed, moderate to strong correlations were reported between MA and general trait anxiety (Betz, 1978; Hembree, 1988, 1990; Hart and Ganley, 2019). It was also demonstrated that general anxiety and MA development are predicted by common genetic and individual-specific environmental risk factors (Wang et al., 2014). Both kinds of anxiety present common cognitive biases, such as the tendency to shift the attention towards emotionally negative stimuli (i.e., numbers; Rubinsten et al., 2015). However, MA and general anxiety are considered to be distinct constructs, with correlations between different measures of MA being stronger than between MA and general anxiety, (for review, see, Ashcraft and Ridley, 2005; Dowker et al., 2016). Importantly, compared to males, females report higher levels of general anxiety (e.g., Núñez-Peña and Bono, 2019; Szczygiel, 2020) as well as higher rates of clinical anxiety (for review, see, McLean et al., 2011).

### Neuroticism, Numeracy and Gender Gap. A Possible Connection?

Stable personal dispositions to anxiety, and to emotional instability in general, are associated with the higher-order trait neuroticism (e.g., Soto and John, 2017), one of the personality traits categorized in the Big Five model (Goldberg, 1990; Costa and McCrae, 1992; John et al., 2008). Neuroticism is characterized by sadness, fear, and emotional instability (Marengo et al., 2021). Individuals who are high in this trait tend to fail in coping with frustration, irritability, and stress, and are more reactive and more sensitive to criticism and hostility; in contrast, those who are low tend to be more emotionally stable and resilient (Costa and McCrae, 1992). Higher levels of neuroticism were associated with higher likelihood of manifesting anxiety symptoms (e.g., Sexton et al., 2003; Kotov et al., 2010; Lyon et al., 2021), thus making plausible an association of this trait also with MA. Moreover, neuroticism levels were found to be higher among women than men (Chapman et al., 2007; Weisberg et al., 2011), and this gender difference was reported across countries (Costa et al., 2001; Schmitt et al., 2008). To date, the relationship between MA and neuroticism has not been investigated. However, it is plausible to presume that the two personal dispositions interact, similarly to MA and general anxiety.

There is evidence supporting the predictive role of personality in accounting for individual differences in performance on large-scale competence assessment tests (Borghans et al., 2016; Cerni et al., 2021). Neuroticism, in particular, was found to negatively correlate with performance on various cognitive tasks. Higher levels of neuroticism correlated with lower scores on verbal reasoning (Chamorro-Premuzic et al., 2006; Cerni et al., 2021) and intelligence (Moutafi et al., 2006; Graham and Lachman, 2014), as well as with slower reaction times in response inhibition (Graham and Lachman, 2012). Considering numeracy, neuroticism seems to negatively affect numeracy test performance (Spinath et al., 2010; Rammstedt et al., 2016; Brandt et al., 2020), even if findings are not consistent; indeed, in some studies, no association between numerical skills and neuroticism was documented (Chamorro-Premuzic et al., 2006; Meyer et al., 2019; Cerni et al., 2021).

To sum up, MA and neuroticism were both found to have an impact on numeracy, but results were mixed, especially considering the relationship of neuroticism with numeracy and the MA effect on numeracy according to gender. In our opinion, considering jointly these non-cognitive factors, where males and females tend to consistently show differences, should promote a better understanding of the mechanisms underlying gender differences in numeracy competence.

### The Present Study

Beside gender differences in numeracy and MA, females and males are found to differ also for other aspects of emotional functioning. For instance, females report higher levels of general anxiety (e.g., Núñez-Peña and Bono, 2019; Szczygiel, 2020) and, accordingly, higher levels of neuroticism (Chapman et al., 2007; Weisberg et al., 2011).

With this in mind, we set out to explain the relationship between MA, neuroticism and numeracy, and related gender differences. We advance the proposal that neuroticism level should correlate with MA (as general anxiety does), and so a gender gap in MA may be linked to a gender gap in neuroticism. With an exploratory approach, we proposed that these correlations may differently interact with numeracy performance in males and females.

We tested our hypotheses in a sample of undergraduate STEM students. In this field, females are dramatically underrepresented (e.g., Morana and Sagramora, 2021) and therefore gender differences in numeracy and math-related affects have been largely overlooked.

Although common sense would suggest that MA does not trouble STEM students, there are findings implying the contrary. Even among students that planned careers or majors with extensive mathematical teaching, feelings of uneasiness about math teaching and testing are common (Betz, 1978; Kaleva et al., 2019).

We thus recruited a sample of male and female STEM undergraduates, with equal levels of Intelligent Quotient (IQ), a variable that strongly affects numeracy (e.g., Cerni et al., 2021). We collected measures of MA and neuroticism using self-report questionnaires. Additionally, to measure numeracy, we administered a competence assessment test based on the one developed by the Italian National Agency for the Evaluation of Universities and Research Institutes (Agenzia Nazionale per la Valutazione del Sistema Universitario e della Ricerca, ANVUR).

**Figure 1:**
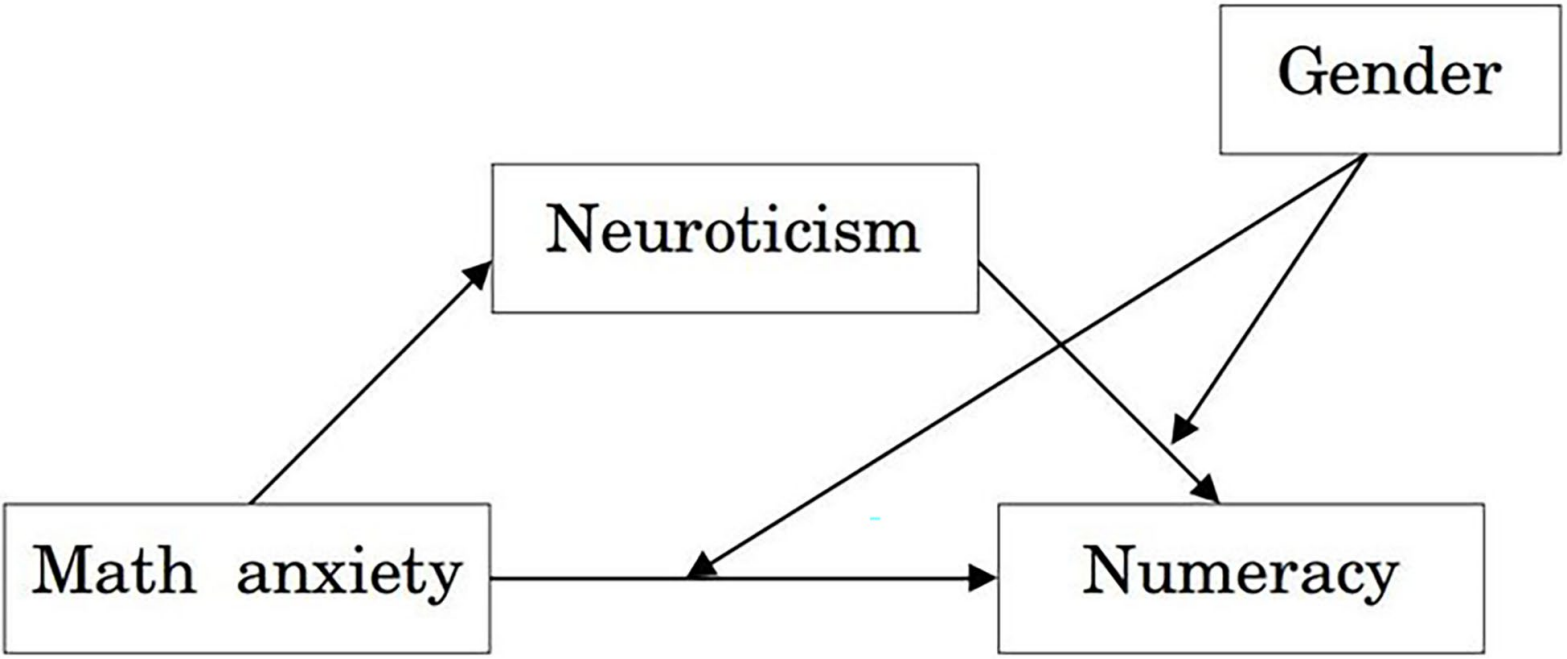
The moderated mediation model tested in the present study. Arrows show the direction of the predicted effects. Neuroticism is the mediator, while gender moderates the path from MA to numeracy e from neuroticism to numeracy.

We tested whether in this sample gender differences would emerge in numeracy, MA and neuroticism scores and whether the non-cognitive factors would be differently related to numeracy in males and females. Based on previous findings, we expected: (1) a gender disparity in numeracy scores, even if the whole sample was familiar with mathematical and numerical contents, (2) higher levels of MA and neuroticism in females than in males, and (3) a stronger association of the non-cognitive variables with numeracy in females than in males. Moreover, we explored a possible pattern of relationships among these variables through a mediation analysis. We examined whether the direct association between MA and numeracy may be partially mediated by neuroticism. In our view, a domain-specific effect, such that of MA on numeracy, may be partly driven by more general personal predisposition, in this case the tendency to be emotional instable. Subsequently, we investigated whether differences between males and females may emerge in this pattern of relationships, by adding gender as a moderator of the influence of the non-cognitive factors on numeracy (see Figure 1 for a graphical representation of the paths).

We believe that investigating these patterns will provide a better understanding about the extent to which non-cognitive skills contribute to numeracy performance among STEM students, who are expected to be more proficient in this competence, and about the possible gender differences in a field where women need to be encouraged.

## Materials and Methods

### Participants

Participants volunteered in the context of an ongoing research project involving freshmen and senior students from Italian universities. Recruitment was restricted to university students aged between 18 and 30 with no history of developmental and/or learning disorders. In the present study, we included students enrolled in STEM undergraduate courses^3^ (*N* = 146, females = 76). Then, we selected a subgroup of females matched to males for IQ (see Tasks and procedure for details on IQ measure). This matching procedure led to a final sample of 140 participants divided into two groups (70 females and 70 males). Moreover, we controlled for group differences in personal characteristics, in particular, age, education (i.e., total years of formal education), math knowledge at university entry (i.e., the final high school grade in mathematics), average grade,^4^ European Transfer and Accumulation System (ECTS) credit points^5^ at the time of the test (as a measure of the learning achievements they accomplished), ECTS points specifically for math and socio-economic status.^6^ Table 1 shows descriptive statistics for each group and corresponding *t*-tests. All the control variables did not statistically differ between the two groups.

**Table 1:** Descriptive statistics of female and male participants.

	Females					Males					*t*	*p*
	*N*	Mean	SD	Min	Max	*N*	Mean	SD	Min	Max		
Age	70	21.4	1.89	19	30	70	21.19	1.64	19	27	0.72	0.475
IQ	70	123.34	8.63	78	128	70	123.66	8.86	76	128	−0.21	0.832
Level of education	70	15.34	0.95	14	16	70	15.23	0.98	14	16	0.70	0.484
Math grade in high school	69^a^	8.41	1.24	6	10	70	8.36	1.26	6	10	0.20	0.845
Average grade at university	66^a^	25.4	2.43	21	30	69^a^	25.65	2.37	20	30	−0.59	0.557
Total ECTS	70	85.46	52.34	0	171	70	91.47	61.07	0	180	−0.63	0.533
ECTS in math	70	33.21	35.76	0	157	70	39.83	42.65	0	159	−0.99	0.322
Socio-economic status	70	4.54	2.05	0	8	70	4.66	1.94	2	8	−0.34	0.736

a Some answers were missing or invalid.

The majority of students were registered at mathematics (*N*_F_ = 12; *N*_M_ = 16), information engineering (*N*_F_ = 9; *N*_M_ = 11), and industrial engineering (*N*_F_ = 7; *N*_M_ = 9). The courses with the highest female ratio were biotechnologies (*N*_F_ = 8; *N*_M_ = 1), food science and technologies (*N*_F_ = 3; *N*_M_ = 1), statistics (*N*_F_ = 8; *N*_M_ = 4) and biology (*N*_F_ = 7; *N*_M_ = 3). The courses with the highest male ratio were chemistry and environmental sciences (*N*_F_ = 1; *N*_M_ = 2) and physics (*N*_F_ = 6; *N*_M_ = 11). Details about the specific degree courses attended by participants divided by gender can be found in the Supplementary Table 1.

Participants gave written informed consent before study participation. After completing the tasks, they received 12 euros as reimbursement for the time they spent regardless of the results obtained. The study was approved by the SISSA Ethics Committee and was conducted according to the guidelines of the Declaration of Helsinki.

### Power Calculation

Statistical power for the mean differences between two groups was estimated with the G*Power statistical software (Faul et al., 2009). With an alpha level of 0.05, a medium effect size can be detected with 140 participants with a power of 0.90. For the mediation analysis, statistical power was estimated with Monte Carlo power analysis for indirect effects through the online software designed by Schoemann et al. (2017). With an alpha level of 0.05 and assuming a correlation of 0.55 between MA and neuroticism (e.g., correlation between MA and general test anxiety, Ganley et al., 2021), of 0.40 between MA and numeracy (e.g., approximate correlation between MA and multiplication problem accuracy in undergraduate students, Hoffman, 2010) and of 0.03 between neuroticism and numeracy (e.g., Cerni et al., 2021), in simple mediation analysis a power of 0.85 can be reached with 140 participants.

### Tasks and Procedure

The study was carried out online from March to July 2021 and was advertised through online channels (Facebook, Sona). Participants received the instructions and the links to the tasks *via* e-mail. The tasks were administered in two separate sections, no more than 2 weeks apart, to prevent participants’ fatigue. The first section included personal information questions (as detailed in the Participant section), the Raven’s Progressive Matrices, the Abbreviated Math Anxiety Questionnaire (AMAS) and the Big Five Inventory (BFI). This section ended with a set of cognitive tasks, which are not part of the present study. The second section included a numeracy test. The order of the tasks was the same for all participants. All the tasks and questionnaires employed in the present work were administered through Google Forms. Details are provided in the below sections.

#### Raven’s Progressive Matrices

A reduced nine-item form of the Raven’s Standard Progressive Matrices was used (Bilker et al., 2012). The individual score on the nine-item scale was transformed in the prediction of the total score on the 60-item scale applying the formula reported by Bilker et al. (2012). The predicted total score was converted into an IQ score according to age-appropriate standardization (Raven, 1938).

#### Big Five Inventory (BFI)

Neuroticism was assessed with the relative items of the BFI (John et al., 1991; Italian version: Ubbiali et al., 2013: English version available online).^7^ This questionnaire consists of 44 items evaluating the traits of the Five-Factor model (agreeableness, conscientiousness, extraversion, neuroticism and openness to experience). Eight of these items assess neuroticism. Each item corresponds to a statement and participants have to rate their agreement on a five-point Likert scale, with 1 indicating “Strongly disagree” and 5 “Strongly agree.” The mean score for trait neuroticism was calculated. In our sample, Chronbach’s alpha for the Neuroticism items was 0.85.

#### Abbreviated Math Anxiety Scale

The Abbreviated Math Anxiety Scale (AMAS)^8^ (Hopko et al., 2003; Italian version: Primi et al., 2014) consists of nine items, each representing a situation regarding mathematical learning or testing, and participants had to indicate on a five-point Likert scale the level of anxiety experienced in that situation (1 = low anxiety; 5 = high anxiety). The AMAS total score was calculated for each participant as the sum of the nine items. Higher scores correspond to high math anxiety levels. Cronbach’s alpha for our sample was of 0.85.

#### Numeracy Test

To measure numeracy competence, we built a reproduction of the TECO-T (TEst of COmpetence), developed by ANVUR (Agenzia Nazionale per la Valutazione del sitema Universitario e della Ricerca), the Italian National Agency for the Evaluation of Universities and Research Institutes, to evaluate students’ generic literacy and numeracy competencies acquired during the university course (Rumiati et al., 2019). The items of the present numeracy test entailed the same structure and the same difficulty level as the TECO-T: an ANVUR member, expert in achievement test development, reviewed the design and the contents of the present test. The numeracy test assessed logical and quantitative thinking skills and its content was not related to curricular teaching. The test included 15 multiple-choice questions, each consisting of a word problem and four response options. The questions are grouped into three modules of five questions each. In the “Graphs and Tables module,” participants had to extract information from a graph (3 items) or a table (2 items) to infer relations between variables. In the “Logical reasoning” items, conclusions had to be drawn based on some numerical or verbal premises. To answer the questions of the “Infographics” module, participants had to infer information from visually represented data concerning the elderly population in Italy. Participants had 40 min to complete the test.^9^ Item score was 1 (right) and 0 (wrong). The sum of correct answers was converted into an accuracy rate as a measure of numeracy competence. The TECO-T was standardized on a representative sample of 827 Italian undergraduate students (see ANVUR, 2016, 2021).

### Data Analysis

All analyses were performed using the free software R v.4.1.1 (R Core Team, 2020).

As a first step, we carried out a set of *t*-tests to investigate gender differences between the variables of interest: neuroticism, MA and numeracy. Moreover, to test whether numeracy is differently related to MA and neuroticism according to gender, we ran Pearson correlation analyses on the whole sample and separately for females and males. We then conducted path analysis-based mediation to explore whether the effect of MA on numeracy is mediated by neuroticism regardless of gender. Subsequently, we performed a moderated mediation analysis (Preacher et al., 2007; Holland et al., 2017), where gender was entered as a moderator in the previous mediation model. The mediation and the moderated mediation analyses were conducted with the PROCESS tool for R (Hayes, 2021). An index of moderated mediation was used to test the significance of the moderated mediation, i.e., the difference of the indirect effects between males and females (Hayes, 2015). The continuous variables were standardized before estimating the models in PROCESS to obtain standardized regression coefficients (*β*) and standardized indirect effect indexes. Indirect mediating effects and the index of moderated mediation were evaluated with 95% confidence intervals (CIs) estimated using bootstrapping with 10,000 samples. If the CI did not contain zero, the indirect effect and the index of moderated mediation were considered statistically significant (Hayes, 2017).

## Results

Descriptive statistics are reported in Table 2. First, males outperformed females in the numeracy test (*t*(138) = 2.49, *p* = 0.014, Cohen’s *d* = −0.42). Second, females scored higher in the MA and neuroticism scales compared to males. The difference between the two groups was statistically significant both for MA (*t*(138) = 3.26, *p* = 0.001, Cohen’s *d* = 0.55) and neuroticism (*t*(138) = 4.62, *p* < 0.001, Cohen’s *d* = 0.78).

**Table 2:** Descriptive statistics and correlations (Pearson’s *r*) between math anxiety (MA), neuroticism and numeracy scores—correlation coefficients.

	*M*	SD	Range	Skewness	Kurtosis	1	2	3
Whole sample (*N* = 140)								
1. MA	19.71	6.56	9.00–43.00	0.69	0.35	1.00		
2. Neuroticism	3.19	0.81	1.50–4.75	−0.12	−0.77	0.32^***^	1.00	
3. Numeracy	0.69	0.16	0.20–1.00	−0.42	−0.17	−0.30^***^	−0.02	1.00
Females (*N* = 70)								
1. MA	21.46	6.13	11.00–35.00	0.48	−0.64	1.00		
2. Neuroticism	3.49	0.71	2.00–4.75	0.01	−0.99	0.21	1.00	
3. Numeracy	0.65	0.15	0.27–0.93	−0.38	−0.49	−0.36^**^	0.25^*^	1.00
Males (*N* = 70)								
1. MA	17.96	6.56	9.00–43.00	1.09	1.88	1.00		
2. Neuroticism	2.9	0.79	1.50–4.25	−0.05	−1.11	0.28^*^	1.00	
3. Numeracy	0.72	0.17	0.20–1.00	−0.48	0.16	−0.17	−0.10	1.00

* *p* < 0.05;

** *p* < 0.01;

*** *p* < 0.001.

*M*, mean; SD, standard deviation.

We inspected relationships between MA, neuroticism, and numeracy scores in the total sample and separately for both genders through zero-order Pearson correlation (Table 2). When the whole sample was considered, MA was positively associated with neuroticism and negatively with numeracy. When the two groups were considered separately, MA was positively associated with neuroticism only in males, and negatively associated with numeracy only in females. Additionally, in females, the numeracy score was positively correlated with neuroticism.

To explore whether neuroticism mediates the relationship between MA and numeracy, the indirect and direct effects were calculated. Table 3 reports detailed results. The total effect (*c*) and the direct effect (path *c′*) of MA on numeracy were significant and moderate, as well as the association of MA with neuroticism (path *a*). The association of neuroticism to numeracy (path *b*) and the indirect effect of MA on numeracy (*ab*) were not significant, indicating that neuroticism did not mediate the effect of MA on numeracy. We further investigated gender differences by adding gender as a moderator of the effects of MA and neuroticism on numeracy (paths *b* and *c′*, see Figure 1 for schematic representation). The interaction between gender and MA was not statistically significant: for both males and females the association between MA and numeracy (conditional path *c′*) was negative. So, gender cannot be identified as a moderator of the relationship between MA and numeracy.

**Table 3:** Results of simple mediation and moderated mediation.

	*β*	*p*	CI	*R* ^2^
**Mediation**				
Outcome: neuroticism (mediator)				0.10
MA (*a*)	0.32	<0.001	0.16–0.48	
Outcome: numeracy (dependent variable)				0.09
MA (*c’*)	−0.32	<0.001	−0.49 –– 0.15	
Neuroticism (*b*)	0.08	0.364	−0.09–0.25	
Total effect (*c*)	−0.30	<0.001	−0.45 –– 0.13	
Indirect effect (*ab*)	0.03		−0.02–0.08	
				
**Moderated mediation**				
Outcome: Neuroticism (mediator)				0.10
MA (*a*)	0.32	<0.001	0.16–0.48	
Outcome: numeracy (dependent variable)				0.16
MA	−0.15	0.196	−0.38–0.08	
Neuroticism	−0.07	0.580	−0.30–0.17	
Gender (female = 1)	−0.37	0.034	−0.71 –– 0.03	
MA × gender (moderation of *c′*)	−0.28	0.103	−0.61–0.06	
Neuroticism × gender (moderation of *b*)	0.43	0.015	0.08–0.77	
Conditional effect of mediator (*b*)				
Male	−0.07	0.580	−0.30–0.17	
Female	0.36	0.006	0.10–0.62	
Conditional direct effect (*c′*)				
Male	−0.15	0.196	−0.38–0.08	
Female	−0.43	<0.001	−0.67 –– 0.19	
Conditional indirect effect (*ab*)				
Male	−0.02		−0.10–0.06	
Female	0.12		0.04–0.21	
Index of moderated mediation	0.14		0.03–0.27	

Letters in italics refer to the traditional representation of mediation as a path diagram (Baron and Kenny, 1986): *a*, path from the predictor (i.e., math anxiety) to the mediator (i.e., neuroticism); *b*, path from the mediator to the dependent variable (i.e., numeracy); *c′*, direct path from the predictor to the dependent variable, when the effect of the mediator is accounted for; *ab*, indirect path of the predictor on the dependent variable through the mediator; *c*, total effect of the predictor on the dependent variable (*c = ab + c′*).

On the other hand, the interaction between gender and neuroticism was significant. Accordingly, females’ standardized regression coefficient for path *b* indicate a significant moderate positive association between neuroticism and numeracy, while for males this association had the opposite sign and was not significant. These results identify gender as a moderator of the relationship between neuroticism and numeracy. The test of the indirect effect (conditional *ab*) showed that neuroticism mediated the effect of MA on numeracy in females but not in males. The index of moderated mediation supported the significance of the moderated mediation.

We repeated the mediation and moderated mediation analyses adding the control variables as covariates and we found that the results are fully aligned with those described above. Details are reported in Supplementary Table 2.

## Discussion

In the present study, we investigated the extent to which MA and neuroticism are associated with numeracy and whether there are gender differences in these relationships in STEM undergraduate students. In our sample, males and females were matched for IQ, a factor that strongly predicts numeracy competence (e.g., Cerni et al., 2021). Furthermore, the two groups were balanced for other relevant variables, such as age, education, mathematical knowledge, average grade, general and math learning achievement and socioeconomic status.

Consistently with previous studies, *t*-test statistics confirmed that females scored worse than males on numeracy and showed higher levels of MA and neuroticism. These findings confirmed the established gaps in numeracy, MA and neuroticism. In addition, only females’ numeracy scores significantly correlated with MA (*r* = −0.36) and neuroticism (*r* = 0.25). Second, we explored a mediation model, in which neuroticism mediated the negative path from MA to numeracy. In line with recent meta-analyses (Zhang et al., 2019; Barroso et al., 2021), higher levels of MA were consistently associated with lower numeracy scores in the whole sample, with one standard deviation increase in MA associated with a 0.32 standard deviation decrease in numeracy, but no mediating effect of neuroticism emerged. However, when we added the moderating effect of gender to the model, neuroticism emerged as a significant mediator for females only. Notably, in females MA was positively associated with neuroticism, which in turn had a positive and moderate association with numeracy: the increase of one standard deviation in MA was associated with a numeracy increase of 0.12 standard deviations through the indirect effect of neuroticism. In males, no mediation effect of neuroticism was found, while the relationship between MA and numeracy was not significant.

Even though the general view is that neuroticism has a negative impact on performance, (e.g., Chamorro-Premuzic et al., 2006; Moutafi et al., 2006; Spinath et al., 2010; Graham and Lachman, 2012, 2014; Rammstedt et al., 2016; Brandt et al., 2020; Cerni et al., 2021), there is also evidence that higher levels of this trait can have positive effects. In one study, participants higher in neuroticism were more accurate than those lower in neuroticism on a conflict recognition task (Smillie et al., 2006). In another study, participants higher in the withdrawal facet of neuroticism—a sub-component more related to anxiety - improved their performance on a visual search task as it became more demanding, whereas the opposite was observed in participants with higher volatility facet levels,—a sub-component more related to the expression of negative affect (Van Doorn and Lang, 2010).

Similar evidence was observed in more ecological contexts. Research in organizational psychology showed that higher levels of neuroticism favored higher performance in terms of job productivity during busy workdays (Study 2, Smillie et al., 2006). The relationship between neuroticism level and job performance appeared to be curvilinear rather than linear, suggesting that the positive influence of neuroticism on performance holds up to a certain level of the trait, beyond which such association flattens (Uppal, 2017). Importantly, in higher education neuroticism was also found to have a positive effect on performance. McKanzie and collaborators (McKenzie, 1989; McKenzie et al., 2000) selected students with a specific coping factor, i.e., the (Cattell et al., 1970) independence trait, which entails aspects of dominance, independent mind and innovation. They found that, among these students, neuroticism positively correlated with achievement, and that this positive association was more likely to be observed in more advanced stages of higher education and in study programs with more rigorous assessment procedures.

Taken together, these findings suggest that higher levels of neuroticism positively boost performance, especially when task demands are high and hence individuals likely experience distress. Adjusting the affective state to stable personality characteristics can be an effective emotional regulation strategy (e.g., Tamir and Gutentag, 2017; Hemenover and Harbke, 2020). In this vein, when individuals with higher levels of neuroticism experience unpleasant feelings, they are in the condition of consistency between a transient affective state and what they typically feel according to their personality: this condition prevents the emotional state from disrupting cognitive resources, leading to improved task performance (Tamir and Robinson, 2004; Tamir, 2005).

According to the definition of MA, individuals with higher levels of MA are more likely to experience negative emotions than individuals with lower levels when they perform a numeracy test. It is possible that females reporting higher levels of MA and higher levels of neuroticism activate emotional regulation strategies to tune their momentary affective state (i.e., the negative emotions towards the numeracy test) to their personality, thus enhancing their numeracy performance. To reveal the presence of these regulation strategies, future studies should be targeted to measure state anxiety and affective states during numeracy tasks.

Despite the presence of this positive mediation of neuroticism on the association between MA and numeracy, in our study females’ numeracy competence was still lower than males’. Probably, the small positive indirect effect of MA through neuroticism, even if statistically significant, was not enough for females to overcome the numeracy gap. The regression model with the moderation of gender on MA and neuroticism explained only 16% of the variance in numeracy scores, suggesting that likely other factors are involved. Understanding why women in STEM are still affected by trait MA differently from men should be a future direction of targeted studies to disclose possible causes of the numeracy gender gap.

As for the association between MA and neuroticism, to our knowledge, this is the first time that it has been investigated. Based on previous studies assessing the association between MA and general anxiety (Betz, 1978; Hembree, 1988, 1990; Hart and Ganley, 2019), one would expect that higher levels of MA correspond to higher levels in trait neuroticism, and this is what we observed. Despite this association, MA and neuroticism differ conceptually, as the former refers to a specific situation or activity, and the latter to a general pattern of feelings. Here we provided some evidence in favor of the conceptual independence of MA. First, the magnitude of the correlation between MA and neuroticism was weak-to-moderate (*r* = 0.32). Second, MA and neuroticism correlations with females’ numeracy scores had opposite signs: the correlation was negative for MA and positive in the case of neuroticism. Third, while in the whole sample the effect of MA on numeracy was not mediated by neuroticism, in the female subgroup, where the partial mediation of neuroticism was significant, the effect of MA on numeracy was still strong. This suggests that MA is a domain-specific anxiety trait, which can negatively influence numerical performance beyond general sensitivity to stress.

This study presents some limitations. First, the sample included in the present study is relatively small. Our findings showed significant correlations of numeracy with neuroticism and MA, and a significant indirect effect, for females only. However, we cannot exclude the possibility that with a larger sample significant effects may have emerged for males as well. Moreover, our sample was composed of STEM undergraduate students, with intensive mathematical and scientific training. This prevents the generalization of our conclusions to the other student groups. Further research including students from arts and humanities is needed to understand whether what we observed here could be applied to the whole population or only to the students that are more predisposed towards mathematics and science. Future studies should also include specific measures of general anxiety, such as trait and test anxiety, to identify the extent of the contribution of this specific factor compared to the broader neuroticism trait.

To conclude, understanding the relationship between cognitive and non-cognitive factors can help in planning effective strategies to sustain students in their studies, especially females enrolled in STEM programs. Female students survived the “bottleneck” represented by the university choice, which deprives the scientific field of young females (Ceci et al., 2014). Further in the pipeline, women are even less represented, suggesting that they face more obstacles than men in their careers. We encourage policymakers and educators to consider the role of MA and neuroticism in the context of STEM education. Although students enrolled in this field are supposedly better at mastering numeracy, performance on numeracy tests can still correlate with MA, especially among women, with neuroticism playing a positive role.

## Data Availability Statement

The raw data supporting the conclusions of this article will be made available by the authors, without undue reservation.

## Ethics Statement

The studies involving human participants were reviewed and approved by SISSA Ethic Committee, International School for Advanced Science (SISSA). The patients/participants provided their written informed consent to participate in this study.

## Author Contributions

All authors contributed to the conception and design of the study. ML organized the database. ML and TC performed the statistical analysis and wrote the first draft of the manuscript. All authors contributed to manuscript revision, read, and approved the submitted version.

## Conflict of Interest

The authors declare that the research was conducted in the absence of any commercial or financial relationships that could be construed as a potential conflict of interest.

## Publisher’s Note

All claims expressed in this article are solely those of the authors and do not necessarily represent those of their affiliated organizations, or those of the publisher, the editors and the reviewers. Any product that may be evaluated in this article, or claim that may be made by its manufacturer, is not guaranteed or endorsed by the publisher.

## Footnotes

^1^Official website: https://www.oecd.org/pisa/

^2^Official website: https://www.oecd.org/skills/piaac/

^3^The identification of undergraduate courses as STEM was based on the official classification made by the Italian Ministry of University and Research (Decree Law 942/2020, Attachment A).

^4^In the Italian university system, the exam grades range from a minimum of 18/30 to a maximum of 30/30 (with or without laude).

^5^60 ECTS are equivalent to a full year of study. A Bachelor degree consists of 180 ECTS (European Commission, 2015).

^6^This corresponds to the parents’ highest level of completed education, based on the International Standard Classification of Education (ISCED).

^7^ https://www.ocf.berkeley.edu/~johnlab/bfi.htm

^8^AMAS was provided by the Italian standardization authors after request.

^9^We used the Apps Script tool in Google Form to build a function that forces the form to close after 40 min.
