# Supplementary material for: Numeracy Gender Gap in STEM Higher Education: The Role of Neuroticism and Math Anxiety
Source: Front Psychol. 2022 May 26;13:856405. doi: 10.3389/fpsyg.2022.856405 (PMC9204305; doi:10.3389/fpsyg.2022.856405)
Supplement: Supplementary file 1 [file Table_1.DOCX]

Supplementary Material

**Supplementary Table 1.** Number of participants attending the different STEM degree courses divided by gender.

| **Degree** | **Female** | **Male** |
| --- | --- | --- |
| Agrarian and forestry sciences | 0 | 1 |
| Architecture | 0 | 1 |
| Biology | 7 | 3 |
| Biotechnologies | 8 | 1 |
| Chemistry | 1 | 2 |
| Cultural heritage conservation and restoration | 0 | 1 |
| Environmantal sciences | 1 | 2 |
| Environmental and civil engineering | 1 | 0 |
| Exercise and sport sciences | 3 | 2 |
| Food science and technology | 3 | 1 |
| Geology | 0 | 1 |
| Industrial engineering (e.g. energy, aerospatial, management engineering | 7 | 9 |
| Informatics | 2 | 4 |
| Information engineering (e.g. informatics and biomedic engineering) | 9 | 11 |
| Mathematics | 12 | 16 |
| Pharmaceutical sciences | 1 | 0 |
| Physics | 6 | 11 |
| Statistics | 8 | 4 |
| Zootechnics | 1 | 0 |

**Supplementary Table 2**. Results of simple mediation and moderated mediation when control variables are included as covariates (*N* = 134^a^).

|  |  |  |  |  |
| --- | --- | --- | --- | --- |
|  | *β* | *p* | CI | *R^2^* |
| **Mediation** |  |  |  |  |
| Outcome: Neuroticism (mediator) |  |  |  | 0.16 |
| Age | 0.001 | .994 | -0.28 – 0.28 |  |
| Socio-economic status | -0.12 | .156 | -0.29 – 0.05 |  |
| Math grade in high school | 0.13 | .221 | -0.08 – 0.33 |  |
| Average grade in university | -0.02 | .802 | -0.22 – 0.17 |  |
| Total ECTS | -0.18 | .332 | -0.55 – 0.19 |  |
| ECTS in math | -0.06 | .537 | -0.29 – 0.15 |  |
| Level of education | 0.31 | .086 | -0.04 – 0.66 |  |
| MA (*a*) | 0.36 | <.001 | 0.18 - 0.54 |  |
| Outcome: numeracy (dependent variable) |  |  |  | 0.14 |
| Age | -0.09 | .512 | -0.37 – 0.18 |  |
| Socio-economic status | -0.10 | .245 | -0.26 – 0.07 |  |
| Math grade in high school | 0.05 | .606 | -0.15 – 0.25 |  |
| Average grade in university | 0.03 | .723 | -0.16 – 0.22 |  |
| Total ECTS | -0.09 | .640 | -0.45 – 0.28 |  |
| ECTS in math | 0.25 | .021 | 0.04 – 0.46 |  |
| Level of education | 0.07 | .680 | -0.28 – 0.42 |  |
| MA (*c’*) | -0.21 | .030 | -0.40 - -0.02 |  |
| Neuroticism (*b*) | 0.03 | .74 | -0.14 – 0.20 |  |
| Total effect (*c*) | -0.19 | <.029 | -0.37 - -0.02 |  |
| Indirect effect (*ab*) | 0.01 |  | -0.05 – 0.07 |  |
|  |  |  |  |  |
| **Moderated mediation** |  |  |  |  |
| Outcome: Neuroticism (mediator) |  |  |  | 0.16 |
| Age | 0.001 | .994 | -0.28 – 0.28 |  |
| Socio-economic status | -0.12 | .156 | -0.29 – 0.05 |  |
| Math grade in high school | 0.13 | .221 | -0.08 – 0.33 |  |
| Average grade in university | -0.02 | .802 | -0.22 – 0.17 |  |
| Total ECTS | -0.18 | .332 | -0.55 – 0.19 |  |
| ECTS in math | -0.06 | .537 | -0.29 – 0.15 |  |
| Level of education | 0.31 | .086 | -0.04 – 0.66 |  |
| MA (*a*) | 0.36 | <.001 | 0.17 - 0.54 |  |
| Outcome: numeracy (dependent variable) |  |  |  | 0.21 |
| Age | -0.10 | .466 | -0.37 – 0.17 |  |
| Socio-economic status | -0.09 | .261 | -0.26 – 0.07 |  |
| Math grade in high school | 0.06 | .563 | -0.14 – 0.25 |  |
| Average grade in university | 0.04 | .659 | -0.15 – 0.23 |  |
| Total ECTS | -0.14 | .447 | -0.50 – 0.22 |  |
| ECTS in math | 0.26 | .016 | 0.05 – 0,46 |  |
| Level of education | 0.13 | .459 | -0.22 – 0.48 |  |
| MA | -0.09 | .472 | -0.33 – 0.15 |  |
| Neuroticism | -0.07 | .533 | -0.31 – 0.16 |  |
| Gender (female = 1) | -0.44 | .014 | -0.79 - -0.09 |  |
| MA x gender (moderation of *c’*) | -0.19 | .287 | -0.53 – 0.16 |  |
| Neuroticism x gender (moderation of *b*) | 0.38 | .034 | 0.03 – 0.74 |  |
| Conditional effect of mediator (*b*) |  |  |  |  |
| Male | -0.07 | .533 | -0.31 – 0.16 |  |
| Female | 0.31 | .023 | 0.04 – 0.57 |  |
| Conditional direct effect (*c’*) |  |  |  |  |
| Male | -0.09 | 0.471 | -0.32 – 0.15 |  |
| Female | -0.27 | .048 | -0.54 - -0.003 |  |
| Conditional indirect effect (*ab*) |  |  |  |  |
| Male | -0.03 |  | -0.13 – 0.06 |  |
| Female | 0.11 |  | 0.03 – 0.23 |  |
| Index of moderated mediation | 0.14 |  | 0.01 – 0.30 |  |

*Note. ^a^* 6 participants were removed from the analysis because of missing data.
